# Supplementary material for: Causal Relationship between Aging and Anorexia Nervosa: A White-Matter-Microstructure-Mediated Mendelian Randomization Analysis
Source: Biomedicines. 2024 Aug 16;12(8):1874. doi: 10.3390/biomedicines12081874 (PMC11351342; doi:10.3390/biomedicines12081874)
Supplement: Supplementary file 1 [file biomedicines-12-01874-s001.zip › Supplementary Figure/Figure S4/Singe SNP plot of MR analysis for TL to ubm-b-1635.pdf]

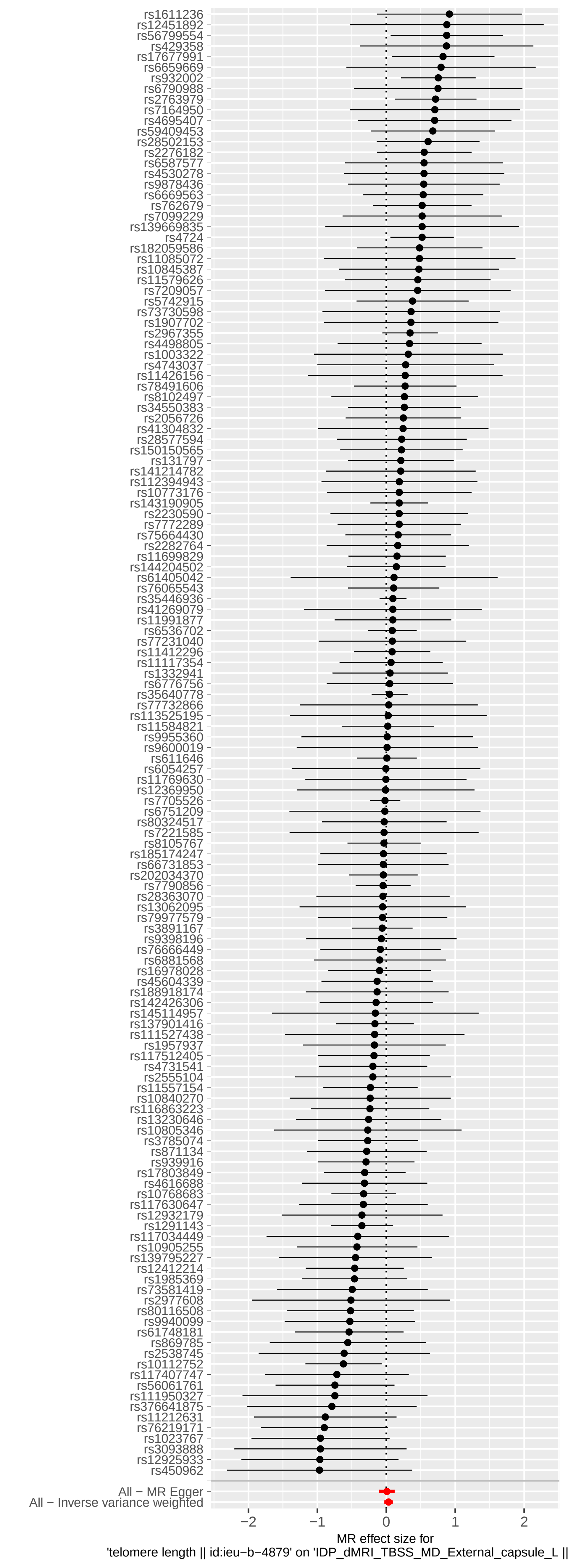
rs1611236
rs12451892
rs56799554
rs429358
rs17677991
rs6659669
rs932002
rs6790988
rs2763979
rs7164950
rs4695407
rs59409453
rs28502153
rs2276182
rs6587577
rs4530278
rs9878436
rs6669563
rs762679
rs7099229
rs139669835
rs4724
rs182059586
rs11085072
rs10845387
rs11579626
rs7209057
rs5742915
rs73730598
rs1907702
rs2967355
rs4498805
rs1003322
rs4743037
rs11426156
rs78491606
rs8102497
rs34550383
rs2056726
rs41304832
rs28577594
rs150150565
rs131797
rs141214782
rs112394943
rs10773176
rs143190905
rs2230590
rs7772289
rs75664430
rs2282764
rs11699829
rs144204502
rs61405042
rs76065543
rs35446936
rs41269079
rs11991877
rs6536702
rs77231040
rs11412296
rs11117354
rs1332941
rs6776756
rs35640778
rs77732866
rs113525195
rs11584821
rs9955360
rs9600019
rs611646
rs6054257
rs11769630
rs12369950
rs7705526
rs6751209
rs80324517
rs7221585
rs8105767
rs185174247
rs66731853
rs202034370
rs7790856
rs28363070
rs13062095
rs79977579
rs3891167
rs9398196
rs76666449
rs6881568
rs16978028
rs45604339
rs188918174
rs142426306
rs145114957
rs137901416
rs111527438
rs1957937
rs117512405
rs4731541
rs2555104
rs11557154
rs10840270
rs116863223
rs13230646
rs10805346
rs3785074
rs871134
rs939916
rs17803849
rs4616688
rs10768683
rs117630647
rs12932179
rs1291143
rs117034449
rs10905255
rs139795227
rs12412214
rs1985369
rs73581419
rs2977608
rs80116508
rs9940099
rs61748181
rs869785
rs2538745
rs10112752
rs117407747
rs56061761
rs111950327
rs376641875
rs11212631
rs76219171
rs1023767
rs3093888
rs12925933
rs450962
All − MR Egger
All − Inverse variance weighted
−2
−1
0
1
2
MR effect size for
'telomere length || id:ieu−b−4879' on 'IDP_dMRI_TBSS_MD_External_capsule_L ||
